# Real-world data on Len/Dex combination at second-line therapy of multiple myeloma: treatment at biochemical relapse is a significant prognostic factor for progression-free survival

**DOI:** 10.1007/s00277-018-3361-2

**Published:** 2018-05-13

**Authors:** Eirini Katodritou, Marie-Christine Kyrtsonis, Sosana Delimpasi, Despoina Kyriakou, Argiris Symeonidis, Emmanouil Spanoudakis, Georgios Vasilopoulos, Achilles Anagnostopoulos, Anna Kioumi, Panagiotis Zikos, Anthi Aktypi, Evangelos Briasoulis, Aikaterini Megalakaki, Panayiotis Repousis, Ioannis Adamopoulos, Dimitrios Gogos, Maria Kotsopoulou, Vassiliki Pappa, Eleni Papadaki, Despoina Fotiou, Eftychia Nikolaou, Evlambia Giannopoulou, Eleftheria Hatzimichael, Nikolaos Giannakoulas, Vassiliki Douka, Kyriaki Kokoviadou, Despoina Timotheatou, Evangelos Terpos

**Affiliations:** 1 Department of Hematology, Theagenion Cancer Hospital, Thessaloniki, Greece; 2 First Department of Propaedeutic Internal Medicine, Laikon General Hospital, National and Kapodistrian University of Athens, Athens, Greece; 3 Department of Hematology and Bone Marrow Transplantation Unit, Evangelismos Hospital, Athens, Greece; 4 Department of Transfusion Medicine, School of Medicine, University of Thessaly, Larissa, Greece; 5 Department of Internal Medicine, Division of Hematology, University of Patras Medical School, Patras, Greece; 6 Department of Hematology, Dimokrition University of Alexandroupolis, Alexandroupolis, Greece; 7 Department of Hematology, School of Medicine, University of Thessaly, Larissa, Greece; 8 Department of Hematology and Bone Marrow Transplantation Unit, George Papanicolaou Hospital, Thessaloniki, Greece; 9 Department of Hematology, Papageorgiou General Hospital, Thessaloniki, Greece; 10 Agios Andreas General Hospital, Patras, Greece; 11 Olympion Clinic, Patras, Greece; 12 Department of Hematology, Faculty of Medicine, School of Health Sciences, University of Ioannina, Ioannina, Greece; 13 Metaxa Cancer Hospital, Pireaus, Greece; 14 Kalamata General Hospital, Kalamata, Greece; 15 Mytilini General Hospital, Mytilene, Greece; 16 Attikon University Hospital, Athens, Greece; 17 University Hospital of Heraklion, Crete, Greece; 18 Medical Affairs, Genesis Pharma SA, Athens, Greece; 19 Department of Clinical Therapeutics, School of Medicine, National and Kapodistrian University of Athens, Athens, Greece

**Keywords:** Lenalidomide-dexamethasone, Biochemical relapse, Symptomatic relapse, Prognosis

## Abstract

We evaluated progression-free survival (PFS) rate of patients treated with lenalidomide/dexamethasone (Len/Dex), the efficacy of the combination, and the prognostic significance of treatment at biochemical vs. clinical relapse on PFS in 207 consecutive myeloma patients treated with Len/Dex in second line, according to routine clinical practice in Greece. First-line treatment included bortezomib-based (63.3%) or immunomodulatory drug-based (34.8%) therapies; 25% of patients underwent autologous stem cell transplantation. Overall response rate was 73.4% (17.8% complete response and 23.7% very good partial response); median time to best response was 6.7 months. Overall, median PFS and 12-month PFS rate was 19.2 months and 67.6%, respectively. 67.5% of patients had biochemical relapse and 32.5% had clinical relapse prior to initiation of Len/Dex. Median PFS was 24 months for patients treated at biochemical relapse vs. 13.2 months for those treated at clinical relapse (HR:0.63, *p* = 0.006) and the difference remained significant after adjustment for other prognostic factors. Type of relapse was the strongest prognostic factor for PFS in multivariate analysis. These real-world data confirm the efficacy of Len/Dex combination at first relapse; more importantly, it is demonstrated for the first time outside a clinical trial setting that starting therapy with Len/Dex at biochemical, rather than at clinical relapse, is a significant prognostic factor for PFS, inducing a 37% reduction of the probability of disease progression or death.

## Introduction

Multiple myeloma (MM) is a plasma cell neoplasm with a disease course characterized by relapses, leading eventually to the development of resistant disease. An asymptomatic, premalignant phase precedes the development of symptomatic disease for several years (monoclonal gammopathy of undetermined significance), while factors associated with the bone marrow microenvironment and the malignant plasma cells lead to the eventual development of symptomatic disease requiring therapy, both during the initial phase of the disease and also at the time of disease relapses [1, 2]. In clinical practice, following the relapse of the disease, treatment decisions are based on the type of previous therapy and response, duration of response, toxicities, patient’s condition, and preferences as well as the type of relapse (aggressive vs. indolent relapse) [3, 4]. However, the optimal timing of offering therapy at the time of relapse is still under investigation. The International Myeloma Working Group (IMWG) recommends starting therapy immediately when patients develop symptomatic relapse or have a rapidly rising paraprotein level or extramedullary disease. Yet for patients who only present a biochemical relapse, i.e., a gradual increase in their paraprotein levels with no clinical symptoms or MM-related organ dysfunction, a watch and wait approach with stringent follow-up is recommended and therapy is delayed [4].

Recent data indicate that treatment with Len/Dex before disease symptoms develop in patients with smoldering (asymptomatic) MM is associated with prolonged progression-free survival (PFS), but most importantly with prolonged overall survival (OS) [5, 6]. However, data regarding the clinical significance of early therapeutic intervention in the relapse setting, i.e., initiating therapy to patients that display only biochemical relapse, rather than symptomatic relapse are not available [3, 4].

Based on the data of two large phase 3 pivotal studies, MM-009 and MM-010, Len/Dex has been established as a standard of care for the treatment of patients who relapse after at least one prior line of therapy [7, 8]. Len/Dex has been extensively used in clinical practice during the last years and local data also confirmed its efficacy in second line or beyond [9]. A sub-analysis of MM-009 and MM-010 studies showed that this combination is more effective, in terms of response rates and PFS, when given earlier in the disease course, i.e., in the first relapse of the disease [10]. No data exist regarding specifically the use of Len/Dex at biochemical relapse rather than at clinical relapse. Recently, Len/Dex combination has been used as a standard comparator, and eventually as the backbone for the development of several new regimens including novel agents such as second-generation proteasome inhibitors (carfilzomib and ixazomib) and monoclonal antibodies (daratumumab, elotuzumab) [3].

Currently, data for Len/Dex treatment and potential variations of the combination as second-line treatment in routine clinical practice outside clinical trials is limited. Practices may differ among physicians, centers, and different regions, e.g., the triggering point of initiation of therapy in patients with relapsing disease, the dosing of lenalidomide or dexamethasone, and the duration of therapy all of which may differ from the controlled environment of a clinical trial. The aim of the current, LEGEND study, was to evaluate the clinical outcomes of patients with relapsed and/or refractory MM (RRMM) receiving Len/Dex treatment at first relapse (biochemical vs. clinical) and capture the treatment patterns following progressive disease as part of routine clinical practice in Greece.

## Patients and methods

### Study design

This was a national, multicenter, non-interventional, retrospective, chart review study aiming to evaluate the clinical outcomes of patients with RRMM receiving Len/Dex treatment at first relapse, as well as document the treatment patterns following progressive disease, as part of the routine clinical practice in Greece. The study was conducted in 18 sites consisting of both public and private hospitals. Participating physicians retrieved all requested information from the medical records of the patients with RRMM that fulfilled the study-specific eligibility criteria and documented the relevant data on paper case report forms.

### Study endpoints

The primary objective of the study was to evaluate the 12-month PFS rate in patients treated with Len/Dex at first relapse. Secondary objectives included the evaluation of (1) PFS in patients treated with Len/Dex at first relapse, (2) PFS at first clinical relapse, (3) PFS at first biochemical relapse, (4) OS rate post second-line treatment initiation, (5) time to next treatment (TtNT) (treatment initiation at second relapse) in patients treated with Len/Dex at first clinical relapse, (6) TtNT (treatment initiation at second relapse) in patients immediately treated with Len/Dex at first asymptomatic relapse, (7) TtNT (treatment initiation at second relapse) in patients with an asymptomatic relapse who were not immediately treated with Len/Dex (watch-and-wait monitoring); additionally, the secondary objectives included the depiction of (8) Len/Dex starting doses at first relapse and the reasons for these (9) dose modifications at the time of second relapse (10, 11) management patterns followed (i.e., treatment intensification, switch in treatment) in patients with clinical relapse while being on or off Len/Dex treatment; (12, 13) management patterns followed (i.e., treatment intensification, addition of drug, switch in treatment, watch-and-wait monitoring) in patients that presented a biochemical relapse while being on or off Len/Dex treatment. Finally, the objectives included (14) Len/Dex treatment discontinuations regarding “Short-Runners” (i.e., who discontinued treatment ≤ 3 months after initiation), “Mid-Runners” (3 < treatment duration ≤ 12 months) and “Long-Runners” (> 12 months), and reasons for discontinuations (permanent or temporary).

Definition of clinical relapse was based on the International Myeloma Working Group (IMWG) criteria [11, 12] as follows: (1) development of new soft tissue plasmacytomas or bone lesions on skeletal survey, magnetic resonance imaging, or other imaging (2) definite increase in the size of existing plasmacytomas or bone lesions; a definite increase is defined as a 50% (and at least 1 cm) increase as measured serially by the sum of the products of the cross-diameters of the measurable lesion (3) hypercalcemia (> 11.5 mg/dL; > 2.875 mM/L) (4) decrease in hemoglobin of more than 2 g/dL (1.25 mM) or to less than 10 g/dL (5) rise in serum creatinine by more than or equal to 2 mg/dL (177 mM/L). With regard to biochemical or paraprotein relapse definition, we used the criteria proposed by the IMWG consensus recommendations for the uniform reporting of clinical trials [13] as follows: doubling of the M-component in 2 consecutive measurements separated by less than or equal to 2 months; or an increase in the absolute levels of serum M protein by more than or equal to 1 g/dL, or urine M protein by more than or equal to 500 mg/24 hours, or involved FLC level by more than or equal to 20 mg/dL (plus an abnormal FLC ratio) in 2 consecutive measurements separated by less than or equal to 2 months. This definition determines significant paraprotein relapse and represents the rate of rise or absolute level of increase in M protein at which the panel considered that myeloma therapy should be restarted in relapsing patients in clinical practice, even if signs and symptoms of new end-organ damage are not yet apparent [13]. An absolute level of increase in M protein by more than or equal to 500 mg/dL, or urine M protein by more than or equal to 200 mg/24 h, or involved FLC level by more than or equal to 10 mg/dL (plus an abnormal FLC ratio) in two consecutive measurements separated by less than or equal to 2 months, is characterized as non-significant paraprotein relapse [13].

PFS was defined as the time from initiation of Len/Dex therapy to disease progression or death (regardless of cause of death), whichever occurred first. Overall response rate (ORR) was defined as the proportion of patients achieving partial response (PR) or better, with Len/Dex as second-line treatment. TtNT was defined as the time from initiation of Len/Dex therapy to initiation of third-line treatment or death (whichever occurred first), and time to progression (TTP) was defined as the time from initiation of Len/Dex therapy to disease progression.

### Patients’ enrolment

Eligible patients were identified from the medical records of each site and included those diagnosed with relapsed and/or refractory MM according to the European Bone Marrow Transplantation (EBMT) group [14] or the IMWG [11] criteria, started second-line treatment with Len/Dex between 01 January 2009 and 01 March 2014 and were followed up by the investigator in the second-line therapy setting for a minimum of 12 months, unless the patient had died before 12 months had elapsed. Patients were enrolled consecutively, starting with those who were initiated on second-line treatment on a date closest to or equal to the earlier cutoff date for subject enrolment (i.e., 01 January 2009). Subject participating in interventional clinical studies were excluded. A signed informed consent form for collecting and analyzing medical data pertinent to the objectives of this study was obtained from living patients while the inclusion of deceased patients in the study was permitted under the condition that consent waiver had been granted by the Scientific Committee and/or Administrative Board of the participating sites. The study was designed, conducted, and reported in accordance with the ethical principles laid down in the Declaration of Helsinki 2008 revision.

### Sample size and statistical analysis

Based on the primary endpoint of 12-month PFS rate and on data from the MM-009 and MM-010 trials, in which the PFS of Len/Dex second-line treatment was 14 months [10], with an expected 12-month PFS rate of 50%, an assessment of at least 195 patients was required in order to estimate the aforementioned proportion with an accuracy of approximately ± 0.07.

The analysis of the primary objective of the study included all patients with available data pertaining to response to second-line treatment. Subsets of patients were also created and analyzed for the purposes of secondary endpoint or exploratory analyses. Continuous variables were summarized with the use of descriptive statistics and categorical variables were displayed as frequencies, while comparisons between groups were performed with the Wilcoxon and the chi-square test. The primary endpoint (12-month PFS rate, starting from the date of therapy initiation until the date of progression or death) and the secondary endpoints pertaining to time-to-event estimations (PFS and TtNT) were estimated by the Kaplan-Meier method and in addition as a proportion based on treating PFS as a binary variable (by dichotomizing the response variable, namely whether the event was observed before or after the 12-month cutoff). Differences in the PFS curves between groups were evaluated with the log-rank test. The effect of variables of interest on PFS was assessed through Cox-regression analysis and on the 12-month PFS rate with logistic regression analysis. All the aforementioned statistical tests were two-sided and were performed at a 0.05 significance level. All analyses were performed with the use of SAS v9.3 software.

## Results

Data from 215 patients were recorded between December 2015 and September 2016; eight patients were excluded from the analysis because Len/Dex treatment started outside the predefined time period; therefore, 207 patients from 18 sites were included in the analysis (Table 1).

**Table 1:** Patient characteristics at diagnosis and at start of second line treatment

Parameters		Total sample^a^ (*N* = 207)	Type of relapse at the start of second-line treatment		*p* value^b^
			Biochemical (*N* = 139)	Clinical (*N* = 67)	
Diagnosis					
Age^c^		67.2 (58.5–73.8)	67.7 (58.8–73.2)	65.8 (57.4–74.8)	0.990
Gender *N* (%)	Female	95 (45.9)	71 (51.1)	24 (35.8)	0.040
	Male	112 (54.1)	68 (48.9)	43 (64.2)	
ISS^d^ *N* (%)	Stage I	54 (26.1)	38 (27.3)	16 (23.9)	0.296
	Stage II	74 (35.7)	53 (38.1)	21 (31.3)	
	Stage III	77 (37.2)	46 (33.1)	30 (44.8)	
	Unknown	2 (1.0)	2 (1.4)		
MM^e^ type *N* (%)	IgA	54 (26.1)	39 (28.1)	15 (22.4)	0.700
	IgD	5 (2.4)	4 (2.9)	1 (1.5)	
	IgG	115 (55.6)	73 (52.5)	41 (61.2)	
	IgM	1 (0.5)	0 (0)	1 (1.5)	
	NS^f^	2 (1.0)	1 (0.7)	1 (1.5)	
	κ	15 (7.2)	10 (7.2)	5 (7.5)	
	λ	7 (3.4)	5 (3.6)	2 (3.0)	
	Unknown	8 (3.9)	7 (5)	1 (1.5)	
Bone disease *N* (%)	Yes	131 (63.3)	80 (57.6)	50 (74.6)	0.029
	No	62 (30.0)	48 (34.5)	14 (20.9)	
	Unknown	14 (6.8)	11 (7.9)	3 (4.5)	
Hemoglobin (g/dL)^c^		10.9 (9.3–12.2)	10.9 (9.2–12.2)	10.8 (9.4–12.1)	0.978
Creatinine (mg/dL)^c^		1.0 (0.8–1.3)	1.0 (0.8–1.2)	1.0 (0.8–1.4)	0.324
Creatinine clearance (ml/min)^c^		75.2 (54.1–98.0)	80.7 (56.1–99.1)	65.5 (43.1–90.8)	0.134
eGFR^g^ *N* (%)	< 60	69 (33.3)	45 (32.4)	24 (35.8)	0.734
	60–90	84 (40.6)	55 (39.6)	29 (43.3)	
	≥ 90	50 (24.2)	35 (25.2)	14 (20.9)	
	Unknown	4 (1.9)	4 (2.9)	0	
Serum Calcium (mg/dL) N (%)	< 11	177 (85.5)	121 (87.1)	56 (83.6)	0.194
	≥ 11	23 (11.1)	12 (8.6)	10 (14.9)	
	Unknown	7 (3.4)	6 (4.3)	1 (1.5)	
Beta-2 microglobulin (mg/L) *N* (%)	< 5.5	112 (54.1)	77 (55.4)	35 (52.2)	0.514
	≥ 5.5	59 (28.5)	37 (26.6)	21 (31.3)	
	Unknown	36 (17.4)	25 (18.0)	11 (16.4)	
Treatment	Bortezomib based	131 (63.3)	86 (61.9)	45 (67.2)	0.459
	IMiD based	72 (34.8)	50 (36.0)	21 (31.3)	0.513
Second-line treatment					
Months from diagnosis to initiation of second line treatment^c^		18.0 (8.8–33.1)	17.5 (9.0–30.7)	19.4 (6.9–39.1)	0.758
Age^c^		69.0 (60.6–75.8)	69.5 (61.1–75)	68.1 (59.4–77.1)	0.730
ECOG PS^h^ *N* (%)	0–1	174 (84.1)	119 (85.6)	55 (82.1)	0.852
	2–4	30 (14.5)	20 (14.4)	10 (14.9)	
	Unknown	3 (1.4)	0	2 (3.0)	
Hemoglobin (g/dL)^c^		12.1 (10.6–13.2)	12.2 (10.9–13.2)	10.9 (9.7–13.0)	0.002
Creatinine (mg /dL)^c^		0.9 (0.7–1.2)	0.9 (0.7–1.1)	1.0 (0.8–1.3)	0.080
Creatinine clearance (mL/min)^c^		76.7 (58.0–114.0)	86.6 (60.0–113.9)	69.9 (49.9–125.0)	0.158
eGFR, mL/min/1.73m^2^, *N* (%)	< 60	51 (24.6)	34 (24.5)	17 (25.4)	0.664
	60–90	83 (40.1)	55 (39.6)	28 (41.8)	
	≥ 90	66 (31.9)	48 (34.5)	18 (26.9)	
	Unknown	7 (3.4)	2 (1.4)	4 (6.0)	
Serum calcium (mg/dL) *N* (%)	< 11	195 (94.2)	133 (95.7)	62 (92.5)	0.955
	≥ 11	3 (1.4)	2 (1.4)	1 (1.5)	
	Unknown	9 (4.3)	4 (2.9)	4 (6.0)	
Beta-2 microglobulin (mg/L) *N* (%)	< 5.5	128 (61.80)	91 (65.5)	37 (55.2)	0.146
	≥ 5.5	36 (17.4)	21 (15.1)	15 (22.4)	
	Unknown	43 (20.8)	27 (19.4)	15 (22.4)	

^a^Type of relapse unknown for one patient

^b^Not including unknown cases

^c^Data reported as median (interquartile range)

^d^International staging system

^e^Multiple myeloma

^f^Non-secretory

^g^Estimated glomerular filtration rate

^h^Eastern collaborative oncology group performance status

### Patients’ characteristics at diagnosis and first-line therapy

Baseline characteristics at diagnosis are depicted in Table 1. At the time of initial diagnosis of MM, the median age was 67 years, 54% were males, and most had IgG ΜΜ (55.6%) followed by IgA (26.1%), light chain only (10.6%), IgD (2.4%), non-secretory (1%), and IgM (0.5%) myeloma. The isotype of disease was not available for eight patients (3.9%). Bone disease was present in the majority of patients (131/207, 63.3%), median hemoglobin was 10.9 g/dL, median creatinine and median creatinine clearance was 1.0 mg/dL and 75.2 mL/min, respectively, serum calcium was greater or equal to 11 mg/dL in 23 (11.1%) patients. Beta-2 microglobulin was greater or equal to 5.5 mg/dL in 59 (28.5%) patients. Regarding the international staging system (ISS), 26.1% were ISS-1, 35.7% were ISS-2, and 37.2% were ISS-3. Cytogenetic abnormalities assessed by either classical cytogenetic analysis (karyotype) or fluorescent in situ hybridization (FISH) were available in a limited number of patients (*N* = 39) at diagnosis, of whom deletion of 17p was detected in 8 (20.5%), del13q in 16 (41.0%), t(4;14) in 7 (17.9%), t(14;16) in 1 (2.6%), and amp1q21 in 5 (12.8%) patients. No significant difference was observed in baseline characteristics between patients having biochemical vs. clinical relapse except for gender and bone disease (*p* < 0.05).

First-line therapy included a bortezomib-containing regimen in 63.3% and an immunomodulatory drug in 34.8%. High-dose melphalan with autologous stem cell transplantation (ASCT) was given in 25.1% of the patients. Consolidation post ASCT was given in 26 patients (50% of the transplanted patients) and maintenance in 26.9% of the transplanted patients. Bisphosphonates were used in 158 patients (76.3%). Response to first-line therapy was stringent complete response/complete response (sCR/CR) in 19.8%, very good partial response (VGPR) in 26.6%, and partial response (PR) in 34.3%, with an ORR of 80.7%.

### Patients’ characteristics at first relapse and second-line therapy with Len/Dex

The median time from diagnosis/primary therapy to initiation of the second-line treatment was 18 months (range 2–147 months). The median age at the time of second-line treatment was 69 years; most patients had Eastern Collaborative Oncology Group Performance Status (ECOG PS) 0–1 (174, 84.1%). Median hemoglobin level was 12.1 g/dL, median creatinine and estimated creatinine glomerular filtration (eGFR) were 0.9 mg/dL and 76.7 mL/min, respectively, beta-2 microglobulin was greater or equal to 5.5 mg/L in 36 (17.4%) patients, and serum calcium was greater or equal to 11 mg/dL in 3 (1.4%) patients; in two patients having biochemical relapse, serum calcium was 11.0 and 11.2 mg/dL respectively for reasons not related to MM, still lower than 11.5 mg/dL which is required for symptomatic hypercalcemia, according to the definition of clinical relapse. Data regarding conventional cytogenetics and FISH were available in a very limited number of patients. At the time of initiation of second-line therapy with Len/Dex, 67 patients had a clinical relapse (32.5%) and 139 (67.5%) had a biochemical relapse (the type of relapse was not recorded in one patient; EBMT [14] and IMWG [11] criteria were used in 42 and 164 patients respectively and no statistically significant differences between these two groups were identified with respect to characteristics at either MM diagnosis or at the start of second-line treatment). In patients with a biochemical relapse, the “watch-and-wait” period was up to 1 month in 78 patients (56.1%), 1–2 months in 29 patients (20.9%), 2–3 months in 12 patients (8.6%), and > 3 months in 20 patients (14.4%). Baseline characteristics before second-line treatment between patients treated at biochemical vs. clinical relapse are shown in Table 1. As expected, hemoglobin was significantly lower (*p* = 0.002), whereas serum creatinine and beta-2 microglobulin were higher in patients treated at clinical relapse, though a statistical significance was not reached for the latter two parameters.

Lenalidomide approved starting dose of 25 mg once daily orally on days 1–21 of each 28-day cycle was administered in most of the patients (165, 79.7%); a proportion of patients reasonably received a different starting dose; 27 patients (13%) started at a dose of 10 mg daily, 10 patients (4.8%) at 15 mg every other day, 2 (1.0%) at 5 mg once daily, and 3 (1.4%) at 5 mg every other day mainly due to renal impairment (17 patients, 40.5%), age (8 patients, 19.0%), and presence of leukopenia (6 patients, 14.3%). Most patients received weekly dexamethasone [40 mg/day in 64 patients (30.9%), 20 mg/day in 77 patients (37.2%)], whereas 52 (25.1%) patients received 40 mg/day on days 1–4 and 15–18 of each 28-day cycle for the first four cycles, followed by 40 mg weekly (days 1, 8, 15, and 22 of each 28-day cycle). The most common reason for administering lower doses of dexamethasone was age (70.3% patients).

The median duration of treatment with Len/Dex was more than a year, 13.6 months. In particular, the median duration of treatment with Len/Dex was 17.9 and 9.0 months for patients treated at biochemical and clinical relapse, respectively. Ninety-four patients continue to benefit from Len/Dex treatment for almost 2 years (median 21.5 months) after its initiation. The main reason for the discontinuation of Len/Dex treatment was disease progression (57 patients, 54.8%) and less often toxicity (18 patients, 17.3%). Among the 104 patients that discontinued, 57 (54.8%) received Len/Dex treatment for more than a year, with a median duration of treatment 21.8 months and were classified as “Long-Runners” (> 12 months), 35 (33.7%) stayed on therapy for a median duration of 7.4 months and were classified as “Mid-Runners” (3 < treatment duration ≤ 12 months), whereas 12 (11.5%) were classified as “Short-Runners” (≤ 3 months after initiation). There was no difference in the reasons for discontinuation among those with different durations of treatment.

### Response rates and outcomes after therapy with Len/Dex at first relapse

In all patients, the ORR with Len/Dex was 73.4% (95% CI 67.4–79.4%) with most of the patients achieving a VGPR (31 patients, 15%) or PR (87 patients, 42%) (Table 2); the ORR for patients treated at biochemical was marginally higher compared to those treated at clinical relapse (77.7 vs. 65.7%; *p* = 0.066); M-component levels at relapse was not correlated with ORR (*p* = 0.79). Median time to first documented response was 2.3 months and to best documented response was 6.7 months.

**Table 2:** Response rates of second line treatment

	Total sample^a^ (*N* = 207)	Relapse at the start of second-line treatment		*p* value
		Biochemical (*N* = 139)	Clinical (*N* = 67)	
Response	*N* (%)	*N* (%)	*N* (%)	0.021
sCR^b^	10 (4.8)	6 (4.3)	4 (6.0)	
CR^c^	27 (13.0)	15 (10.8)	12 (17.9)	
VGPR^d^	49 (23.7)	36 (25.9)	13 (19.4)	
PR^e^	66 (31.9)	51 (36.7)	15 (22.4)	
MR^f^/SD^g^	16 (7.7)	12 (8.6)	4 (6.0)	
Other	39 (18.9)	19 (13.7)	19 (28.4)	
ORR^h^	152 (73.4)	108 (77.7)	44 (65.7)	0.066

^a^Type of relapse unknown for one patient

^b^Stringent complete response

^c^Complete response

^d^Very good partial response

^e^Partial response

^f^Minor response

^g^Stable disease

^h^Overall response rate

After a median follow-up of 52.8 months, 131 patients have progressed and 112 have died, so that the median TTP was 23.1 months (95% CI 18.4–30.8, range 0.8–88). Reasons for death were MM disease-related in 69 (61.6%) patients, toxicity in 6 (5.4%) patients, and other reason in 37 (33%).

**Fig. 1:**
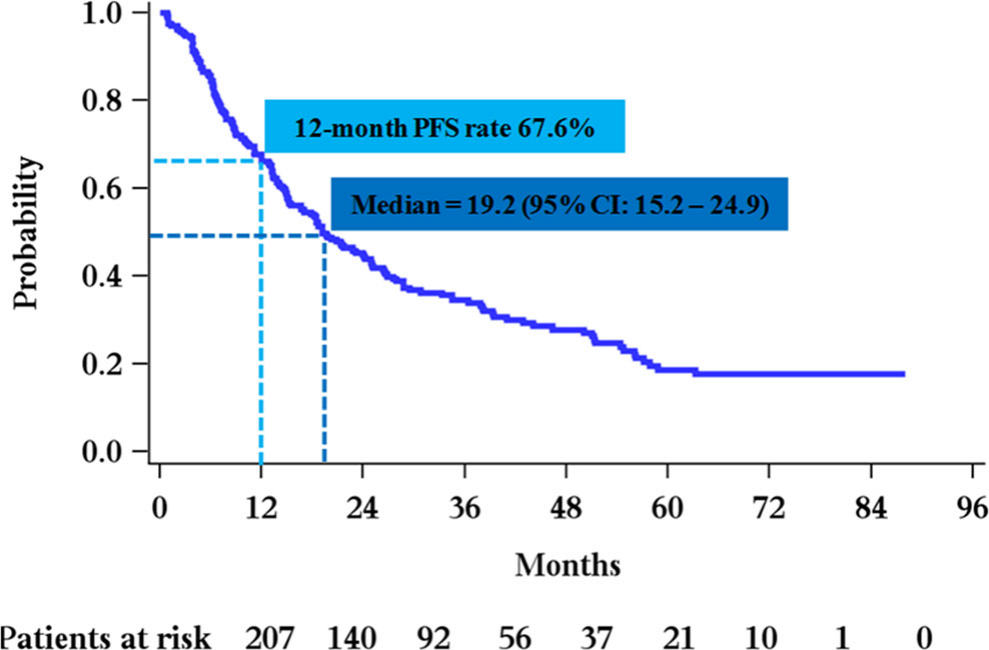
PFS curve for all patients from the initiation of second line treatment (median 19.2 months) and 12-month PFS rate (67.6%)

The 12-month PFS rate for all patients (primary endpoint of the study) was 67.6% (95% CI 60.8–73.5%) (Fig. 1), demonstrating that 137 patients (66.18%, 95% CI 59.74–72.63%) were free of disease progression at 12 months, 68 had experienced disease progression, while two had died without recorded disease progression. In univariate analysis, statistically significant prognostic factors associated with 12-month PFS rate were the type of relapse at the start of second-line therapy (biochemical vs. clinical) (*p* = 0.003) and ISS stage at the time of initial diagnosis (*p* = 0.024). Multivariate analysis for 12-month PFS showed that the type of relapse (*p* = 0.013) and ISS stage (*p* = 0.055) remained statistically significant factors.

**Fig. 2:**
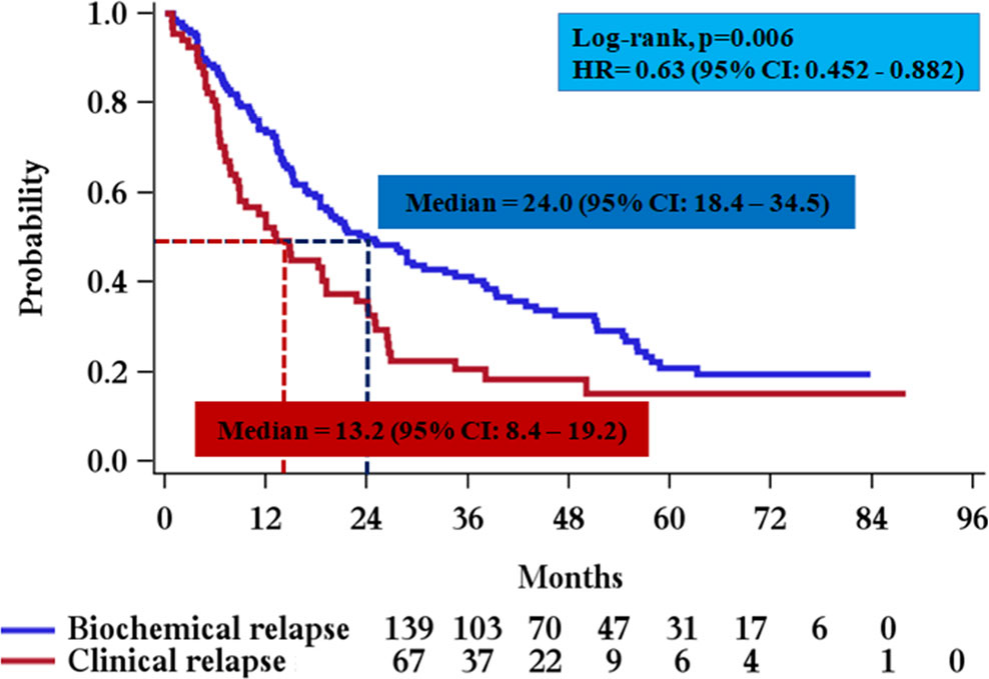
PFS curves for patients who started second line treatment on clinical relapse (median 13.2 months), and for patients that started second line treatment on biochemical relapse (median 24 months)

**Fig. 3:**
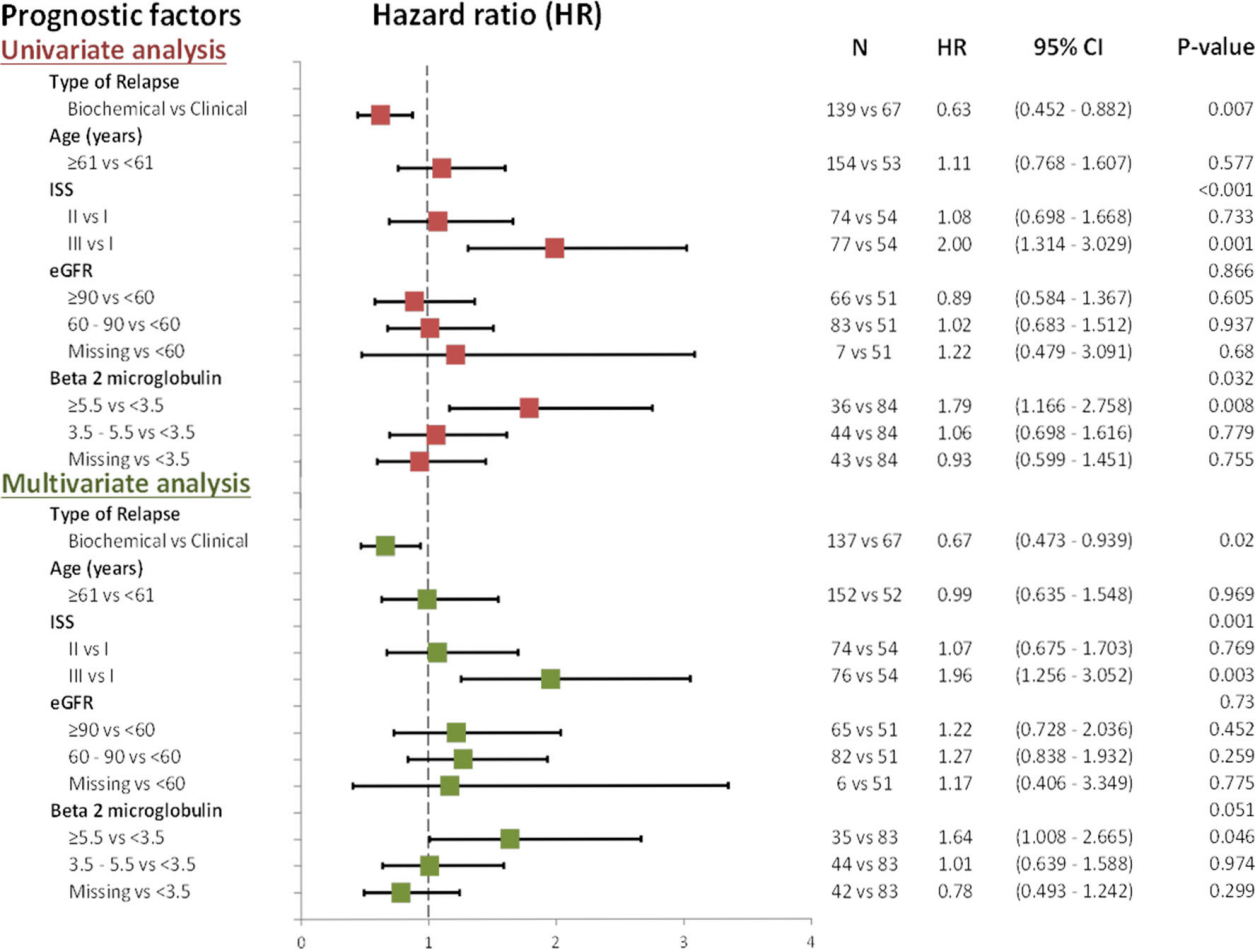
Univariate and multivariate analysis of PFS

The median PFS for all patients in the study was 19.2 months (95% CI 15.2–24.9, range 0.8–88, Fig. 1). After a median follow-up of 52.8 months, 156 events were included in the calculation, 131 of which represented patients whose disease had progressed, and 25 that died without recorded progression. Importantly, approximately 10% of the patients (*N* = 21) remained event free beyond the 5-year follow-up time point, of whom 17 (81%) had biochemical relapse and only 4 had clinical relapse at the time of second-line therapy. For the 67 patients who started second-line treatment on clinical relapse, median PFS was 13.2 months (95% CI 8.4–19.2, range 0.8–88), based on 54 events (41 patients had disease progression and 13 were deceased without recorded progression). For the 139 patients that started second-line treatment on biochemical relapse, the median PFS was 24 months (95% CI 18.4–34.5, range 1.0–83.8) based on 101 events that included 89 disease progression events and 12 deaths without recorded disease progression (Fig. 2). The difference in median PFS for patients treated at biochemical relapse vs. for those treated at clinical relapse was significant (24 vs. 13.2 months, HR:0.63, *p* = 0.006) and remained significant after adjustment for other important prognostic factors, inducing a 37% reduction of the probability of disease progression. Of the 139 patients who started therapy while on biochemical relapse, the majority (119 patients) started treatment within 3 months after the first indication of biochemical relapse and prior to the development of any clinical symptoms and were classified as “immediately treated”. Twenty patients started therapy with Len/Dex after a “watch-and-wait” period beyond 3 months and prior to the development of clinical relapse (“late starters”). The median PFS for “late starters” was 28.7 months (95% CI 15.2–54.3, range 3.9–72,) slightly longer, but not statistically significant compared to “immediate starters”, who had a median PFS of 21.7 months (*p* > 0.05). In univariate analysis, statistically significant prognostic factors for PFS were beta-2 microglobulin at the time of second relapse (*p* = 0.024), the type of relapse (biochemical vs. clinical) at start of second-line therapy (*p* = 0.0052) and ISS at diagnosis (*p* = 0.0002). The levels of serum M protein before second-line treatment initiation did not significantly affect PFS (*p* = 0.32). In multivariate analysis, ISS stage at diagnosis (*p* = 0.001) and beta-2 microglobulin upon treatment initiation (*p* = 0.051) remained statistically significant factors that were associated with PFS, but the type of relapse was the strongest prognostic factor for PFS (*p* = 0.02), (Fig. 3).

The median TtNT for the 67 patients who started Len/Dex at symptomatic relapse was 14.7 months (95% CI 10.4–24.1, range 0.9–88) with almost half of them (*N* = 34) having initiated a third-line treatment. Patients who started Len/Dex on biochemical relapse had a longer TtNT. “Immediately treated” patients on biochemical relapse (*N* = 119) had a median TtNT of 31.1 months (95% CI 22.0–44.1, range 1.0–84.1) with 61 of them having initiated a third-line treatment. “Late starters” (*N* = 20) that initiated Len/Dex beyond 3 months of biochemical and before clinical relapse had a median TtNT of 31.5 months (95% CI 17.3-NE, range 5.3–72.0,) with 12 of them having proceeded to a third-line treatment.

### Management pattern followed at the time of second relapse

**Fig. 4:**
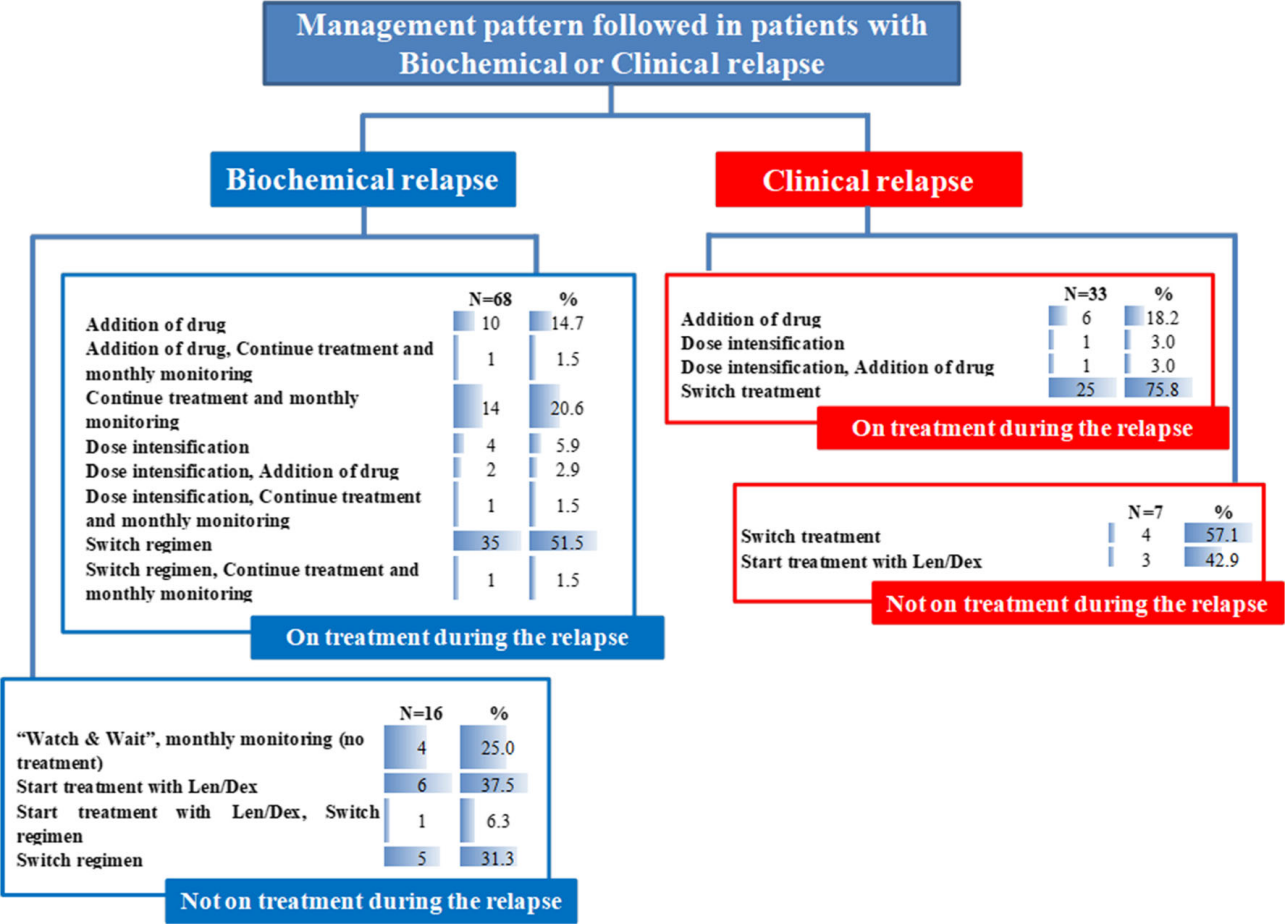
Management pattern followed in patients with Biochemical or Clinical relapse

Figure 4 captures the management pattern followed in 124 patients that relapsed from second-line therapy with Len/Dex. At the time of second relapse, 23 patients were not on treatment, 67 patients were receiving 25 mg of lenalidomide once daily orally on days 1–21 of each 28-day cycle; dexamethasone doses varied but were mainly 40 mg (*N* = 55) to 20 mg (*N* = 31) weekly.

Thirty-three patients had a second clinical relapse while being on treatment with Len/Dex; in 25 (75.8%), the treating physician decided to switch treatment (bortezomib-based in 15 of them) while a third drug was added in 6 patients (18.2%) and the dose was intensified in 2 (6.0%) patients in order to maintain the efficacy of Len/Dex treatment. Among the seven patients with clinical relapse that were not on treatment with Len/Dex, four began a different treatment, and three patients were Len/Dex re-challenged.

In 36 out of 68 patients (53.0%) who experienced a biochemical second relapse while on treatment with Len/Dex, the treating physician decided to switch treatment (bortezomib-based in 19 and pomalidomide in 3); in 14 (20.6%) patients, the treating physician decided to continue treatment with Len/Dex and monitor monthly; in 11 (16.2%) patients, a third drug was added; and in 7 (10.3%) patients, the dose of lenalidomide was intensified aiming to detain the loss of Len/Dex treatment response. Among patients with a biochemical second relapse that were not on treatment with Len/Dex (*N* = 16 patients) in 7 (43.8%) patients, treatment with Len/Dex was re-initiated, while in 4 (25%), a “watch-and-wait” strategy was followed, and in 5 (31.2%), a different treatment started (Fig. 4).

## Discussion

The results of the current national, multicenter, retrospective, chart review study provide a comprehensive representation of the use of Len/Dex combination as a second-line regimen, in the routine clinical practice in Greece, outside the controlled environment of clinical trials. Even though the efficacy and safety of the Len/Dex combination is well documented [7, 8, 10], the current study confirms the effectiveness of the combination in the “real-world” setting focusing on patients who received the combination in their first relapse. Our results are comparable with those of randomized clinical trials in terms of response rates and median PFS [10]. Specifically, the improved outcomes in the LEGEND study may be due to the inclusion of patients treated in the majority at biochemical rather than clinical relapse, the optimal dose adjustments of lenalidomide and mostly of dexamethasone and perhaps the growing experience in the management of toxicities.

Several published studies have shown that patterns of relapse are heterogenous and predict outcome suggesting that therapeutic approach during clinical follow-up should be individualized in order to achieve the maximum benefit [15–17]. Our data show that despite the recommendations that patients with biochemical relapse should be followed rather than treated [4], the majority of practicing physicians preferred to start treating their relapsing patients, while they were still asymptomatic. Importantly, when we compared patients that started therapy at biochemical relapse to those treated at clinical relapse, the median PFS was significantly longer in patients that underwent earlier therapeutic intervention, before the development of symptomatic disease with a 33% reduction in the risk of progression or death. Furthermore, this was translated into a significant difference in the TtNT between the two groups (median 14.7 months for symptomatic relapse vs. 31.1 months for biochemical relapse). Of note, the differences remained significant even after adjustment in multivariate analysis for differences in other important prognostic disease characteristics such as age, ISS, beta-2 microglobulin, and estimated glomerular filtration rate (eGFR) (LDH was highly non-significant and was not further analyzed as a covariate). In addition, first-line treatment administered included first-generation novel agents and it was uniform and did not differ between patients treated at biochemical vs. clinical relapse diminishing thus biases and misleading interpretations induced by therapeutic heterogeneity at diagnosis.

Although there may be lead time bias by starting treatment earlier (i.e., before symptoms develop), data from the Spanish group in elderly patients [18] show that the median time between biochemical and clinical relapse was only 5.1 months (range 2–24), indicating that the difference that was found between those that started therapy at biochemical vs. symptomatic relapse in the LEGEND study can only partly be attributed to this lead time bias. Furthermore, a subgroup of patients (10%) substantially benefited by prolonged therapy with Len/Dex with an event-free survival beyond the 5-year follow-up time point, most of whom (17/21, 81%) had biochemical relapse at the start of second-line therapy, further supporting the potential benefits for earlier rather than later initiation of therapy at first relapse. The results favoring an earlier initiation of therapy at biochemical rather than at symptomatic relapse may also be relevant to the underlying biology of the disease and the mechanism of action of lenalidomide. Thus, during the phase of asymptomatic relapse, the disease burden is probably lower, the disease biology is less aggressive and more responsive to therapy, and the immune system may be more competent and able to control the disease under treatment with immunomodulatory drugs such as lenalidomide. The role of the immune system may be critical in this regard, especially when considering the results of the recent triple combination of Len/Dex with immunotherapy, such as with daratumumab [19] and elotuzumab [20] which show impressive results especially when given at first relapse [21].

Apart from early treatment initiation, equally important is the optimal administration of Len/Dex in second line. The majority of patients received lenalidomide 25 mg once daily orally on days 1–21 of each 28-day cycle as a starting dose, while a proportion received lower doses mainly due to renal dysfunction. This study confirms the importance of lenalidomide 25 mg starting dose and the appropriate dose modifications according to renal insufficiency, supporting the implementation of recommendations that have been made by expert panels [22, 23], in order to fully benefit from the combination. Regarding the dose of dexamethasone, most patients received rather low dose regimens (i.e., weekly doses of 40 mg or less). Therefore, it is not surprising that high-dose dexamethasone (i.e., 40 mg on days 1–4, 9–12, and 17–20) was not used, given the toxicity of this regimen, confirming the data from a prospective study in newly diagnosed patients supporting the use of low rather than high-dose dexamethasone with lenalidomide [24] and a retrospective analysis in patients with relapsed or refractory MM who were treated with lenalidomide and different doses (intermediate and low) of dexamethasone indicated that there was no significant difference in terms of efficacy [25]. Moreover, the use of lower doses of dexamethasone may have improved the outcomes of our patients, by reducing toxicity and early discontinuations; an analysis of the MM-009 and MM-10 studies had also suggested that reduced dexamethasone dose may actually improve treatment efficacy [26]. Regarding the management pattern followed in patients at second-line treatment relapse, switching regimen was the preferred choice by the treating physician in 75% of patients with symptomatic relapse to Len/Dex and in almost half of the patients with biochemical relapse. However, the treating physician tended to add a third agent or to increase the previously adjusted dose of lenalidomide, in order to exhaust the Len/Dex treatment benefit.

Lenalidomide with dexamethasone has recently been approved for the treatment of patients with newly diagnosed MM who are not candidates for ASCT, based on the results of the FIRST study [27]. An increasing number of patients, ineligible for transplant, are thus expected to receive Len/Dex as primary and less likely as second-line therapy. In addition, lenalidomide maintenance has been approved as a monotherapy for patients who have undergone transplantation [28, 29], so it is expected that patterns of therapy will be different when they relapse and Len/Dex combination may be used less often. However, the most important development over the recent years has been the approval of triple combinations with Len/Dex as backbone, in which a third agent, either a proteasome inhibitor (carfilzomib [30] or ixazomib [31]) or a monoclonal antibody (elotuzumab [20] or daratumumab [19]) is added. The results of the LEGEND study set emphatically the issue of timing of initiation of therapy at relapse earlier rather than later, even more so with the availability of more effective therapies for the management of first disease relapse.

The LEGEND study is a retrospective, chart review study and by design has certain limitations. No data regarding the distinction of patients between those that had significant or non-significant biochemical relapse was available [13]. However, the majority of patients were treated according to criteria of significant biochemical relapse as this is the standard practice in Greek Myeloma Study Group centers as well as in other study groups [32]. Fernandez de Larrea [32] presented preliminary results demonstrating that 75% of clinicians decide early treatment at biochemical relapse when this is “significant paraprotein relapse” according to the IMWG Consensus panel criteria [13]. Furthermore, cytogenetics data at first relapse and at initial diagnosis were not available for all patients, so that an analysis of their impact is not possible given the small numbers. A selection bias may have also been introduced but, per protocol, the participating physicians should have included all consecutive patients, not participating in clinical trials, who started therapy in a predefined time period, thus reducing this bias.

In conclusion, the LEGEND study provided a detailed, real-world evaluation of Len/Dex treatment at first relapse and showed that the combination, as second-line treatment, leads to high ORR and prolonged PFS, while patients treated earlier, at biochemical relapse, may have substantial improvement of their outcomes compared to those treated at symptomatic relapse. Treatment with Len/Dex at first relapse has been implemented in the clinical practice as a standard of care, but the results obtained in everyday clinical practice have improved over those in clinical studies, probably due to the earlier treatment decisions at relapse, optimal starting and adjustment doses of lenalidomide and dexamethasone, and improved management of toxicities. Moreover, with the introduction of the combination of several new agents, Len/Dex seals its value as the backbone therapy at first relapse.
